# The Precuneus Region Drives Brain Network Changes in Tremor‐Dominant Parkinson's Disease: Insights from a Morphological Causal Analysis

**DOI:** 10.1002/mco2.70441

**Published:** 2025-10-26

**Authors:** Moxuan Zhang, Siyu Zhou, Pengda Yang, Huizhi Wang, Jinli Ding, Xiaobo Wang, Xuzhu Chen, Chaonan Zhang, Anni Wang, Yuan Gao, Qiang Liu, Yuchen Ji, Yin Jiang, Lin Shi, Chunlei Han, Zhong Yang, Tao Feng, Jianguo Zhang, Fangang Meng

**Affiliations:** ^1^ Beijing Neurosurgical Institute, Beijing Tiantan Hospital Capital Medical University Beijing China; ^2^ Department of Neurosurgery Shandong Cancer Hospital and Institute, Shandong First Medical University and Shandong Academy of Medical Sciences Jinan China; ^3^ Department of Neurosurgery Beijing Tiantan Hospital, Capital Medical University Beijing China; ^4^ Department of Radiology Beijing Tiantan Hospital, Capital Medical University Beijing China; ^5^ Department of Neurosurgery The First Affiliated Hospital of Zhengzhou University Zhengzhou China; ^6^ Department of Humanities and Social Sciences Binzhou Medical University Shandong China; ^7^ Department of Neurology Beijing Tiantan Hospital, Capital Medical University Beijing China

**Keywords:** brain morphology, Granger causality analysis, machine learning, structural covariance networks, Tremor‐dominant Parkinson's disease

## Abstract

The tremor‐dominant (TD) subtype of Parkinson's disease (PD) is characterized by prominent tremor symptoms. However, the temporal and causal relationships between brain structural alterations in TD patients remain unexplored. A total of 61 TD patients and 61 matched healthy controls (HCs) were included in this study. The gray matter volume (GMV) of the bilateral precuneus (PCUN) was significantly reduced in TD patients. A structural covariance network analysis seeded with the left pallidum (PAL.L), which had the most significant differences, revealed a substantial reduction in covariance with precentral gyrus in TD patients. We performed a causal structural covariance network analysis using the TD duration as a pseudotime series. The PCUN, with the highest out‐degree in the cortex, regulates numerous regions, including the supplementary motor area and the extensive temporal lobe. Machine learning was utilized to construct a model that accurately assesses the surgical prognosis based on the above cortical volume and clinical scale, with the aim of assisting in clinical deep brain stimulation (DBS) treatment. These findings suggested a progressive pattern of GMV changes extending from the PAL.L to the PCUN region and continuing to other brain regions, providing insights into the progression of TD and enhancing DBS treatment strategies.

## Graphical abstract

We uncovered a unique morphological progression pattern in tremor‐dominant Parkinson's disease. Based on this, a machine‐learning model combining cortical volume features with clinical assessments was developed to reliably predict deep brain stimulation (DBS) outcomes.

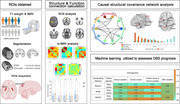

## 1 Introduction

Parkinson's disease (PD), characterized by tremors, akinesia/bradykinesia, and rigidity, is the second most prevalent neurodegenerative disorder following Alzheimer's disease [1]. In 2016, approximately 6.1 million people worldwide were diagnosed with PD. The crude prevalence of PD has grown by about 74% over the past three decades. However, the age‐standardized prevalence exhibited a more gradual increase of 22% [2, 3]. PD is a remarkably heterogeneous disorder, and numerous subtypes have been proposed [4]. Despite the absence of a consensus on methods for distinguishing these subtypes, the most prevalent classification method involves the clustering of clinical symptoms and identification of the dominant symptom. According to this framework, PD can be categorized into the tremor‐dominant (TD) subtype, the akinetic–rigid or postural instability gait disorder (PIGD) subtype, and the intermediate subtype [5]. In patients with the TD subtype, tremors are notably more pronounced than rigidity, and this dominance is often determined through a clinical assessment. The heterogeneity of clinical manifestations presents significant challenges for the diagnosis and treatment of PD. In addition to the clear differences in dominant motor symptoms, patients with the TD subtype exhibit distinct variations in emotional, cognitive, urinary, gastrointestinal, and other autonomic features [6, 7], as well as differences in progression and prognosis [8]. The unique characteristics of the TD subtype underscore the need for research to improve our understanding and address its clinical challenges. These characteristics also suggest the presence of specialized neurological mechanisms inherent to the TD subtype, warranting detailed explorations.

Modern neuroimaging tools have been utilized to further delineate the underlying neural mechanisms involved and better understand the diverse symptoms and progression of PD subtypes. The structural imaging field has introduced two fundamental techniques for quantitative structural image analysis: voxel‐based morphometry (VBM) and surface‐based morphometry (SBM). The VBM analysis provides a comprehensive assessment of gray matter volume (GMV) changes in PD patients compared with healthy controls (HCs). Burton et al. reported extensive gray matter atrophy in PD patients, primarily involving the bilateral temporal and occipital lobes and selected frontal and parietal areas [9]. A study of subcortical nuclei revealed that PD patients had obvious lesions in the bilateral hippocampus, amygdala, thalamus, right caudate nucleus, and putamen [10]. However, the number of studies focusing on PD subtypes remains limited. More comprehensive and methodologically rigorous research on PD subtypes is needed to further elucidate the underlying pathogenic mechanisms.

Recently, the causal structural covariance network (CaSCN) approach, which is based on Granger causality analysis (GCA), has been introduced to assess the causal relationships underlying structural alterations between brain regions [11]. The CaSCN analysis involves constructing a pseudotime series based on disease severity or duration to evaluate GMV progression patterns across the disease course, and has been widely adopted in neuroimaging research [12, 13]. Jiang et al. observed that GMV abnormalities in schizophrenia patients originate in the thalamus and progressively involve the temporal and occipital areas, underscoring the thalamus as a critical hub region [14]. Another study using CaSCN investigated blepharospasm patients and found that the right supplementary motor area was the earliest region affected, with abnormalities progressively involving the cortico‐basal ganglia motor pathway and the visual‒motor integration pathway [15]. Accordingly, we applied CaSCN to characterize the disease‐specific progression of structural alterations in TD.

TD often presents significant clinical challenges, as tremor symptoms typically respond less effectively to conventional dopaminergic treatments than do other motor symptoms, such as bradykinesia and rigidity [16]. Consequently, deep brain stimulation (DBS) has proven to be an effective intervention for managing medication‐resistant tremors in TD patients [16]. Among several potential targets for DBS, the subthalamic nucleus (STN) is most commonly selected because of its well‐documented clinical efficacy. However, the efficacy of DBS often varies between individuals and is challenging to predict prior to surgery. Recently, advances in machine learning have provided promising methods for predicting individualized clinical outcomes based on medical imaging data, offering the potential to optimize DBS treatment strategies [17].

In our study, we used structural magnetic resonance imaging (MRI) to analyze changes in the volumes of the entire cerebral cortex and subcortical nuclei of TD patients, aiming to reveal the unique progression pattern of TD patients at the brain morphology level. Furthermore, we also evaluated the efficacy of DBS and used machine learning to construct a model that could accurately assess the patient prognosis based on the cortical volume and clinical scale scores, aiming to provide assistance for clinical DBS treatment.

## 2 RESULTS

### 2.1 Demographics and Clinical Characteristics

A total of 61 TD patients were enrolled in the study based on the inclusion criteria. Additionally, 61 age‐ and sex‐matched HCs were included, as summarized in Table 1. No statistically significant differences in age or sex were observed between the groups (*p* > 0.05). For the symptom evaluation, all TD patients underwent assessments using the Movement Disorder Society–sponsored revision of the Unified Parkinson's Disease Rating Scale (MDS‐UPDRS), Hamilton Rating Scale for Anxiety (HAM‐A), Hamilton Rating Scale for Depression (HAM‐D), Parkinson's Disease Questionnaire‐39 (PDQ39), Pittsburgh Sleep Quality Index (PSQI), and Voice Handicap Index (VHI) scales, etc.

**TABLE 1:** Demographic and clinical features of the subjects.

Characteristics	TD patients (*n* = 61)	HCs (*n* = 61)	*p* value
Age (years, mean ± standard deviation [SD])	63.36 ± 6.68	62.48 ± 5.49	0.426^a^
Sex (male/female)	35/26	33/28	0.715^b^
Age at disease diagnosis (years, mean ± SD)	55.02 ± 9.17	—	n/a
Duration of the disease (years, mean ± SD)	8.34 ± 5.34	—	n/a
MDS‐UPDRS III score (on‐medication)	45.61 ± 16.47	—	n/a
MDS‐UPDRS III score (off‐medication)	22.49 ± 12.11	—	n/a
MMSE score	26.28 ± 2.27	—	n/a
MoCA score	21.61 ± 3.42	—	n/a
Total LED (mg/day)	831.08 ± 497.87	—	n/a
HAM‐A	14.03 ± 6.99	—	n/a
HAM‐D	15.48 ± 8.47	—	n/a
VHI	28.41 ± 27.80	—	n/a

Abbreviations: HAM‐A, Hamilton Rating Scale for Anxiety; HAM‐D, Hamilton Rating Scale for Depression; HCs, healthy controls; LED, levodopa equivalent dose; MDS‐UPDRS, Movement Disorder Society‐sponsored revision of the Unified Parkinson's Disease Rating Scale; MMSE, Mini‐Mental State Examination; MoCA, Montreal Cognitive Assessment; n/a, not applicable; TD, tremor‐dominant PD; VHI, Voice Handicap Index.

^a^ Mann–Whitney *U* test: TD patients versus HCs.

^b^ Chi‐square test: TD patients versus HCs.

### 2.2 Overall GMV Alterations in TD Patients

**FIGURE 1:**
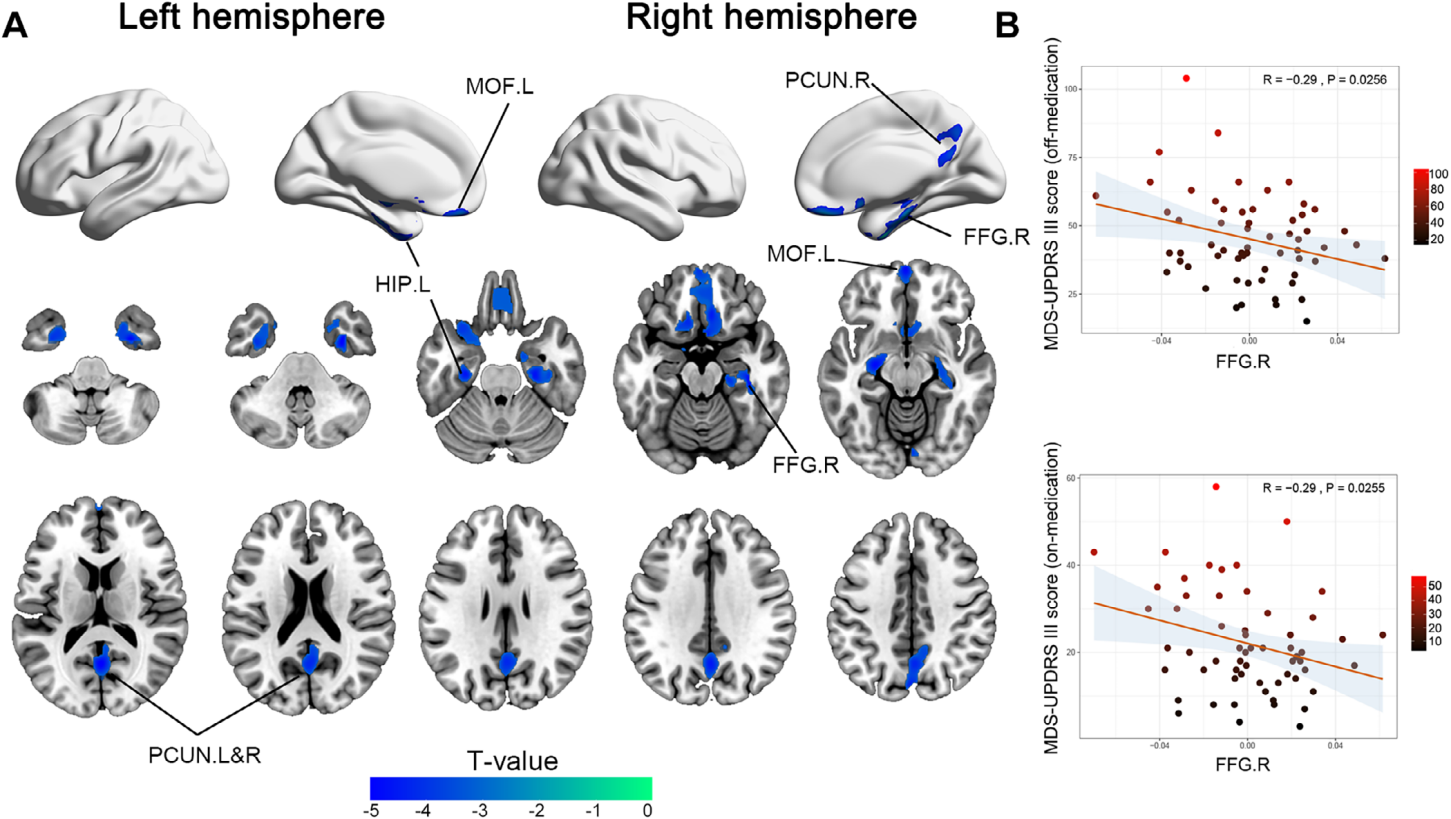
GMV changes in TD patients compared with HCs. (A) Brain regions displaying significant GMV changes in patients with TD compared with HCs are illustrated. The blue areas indicate reductions in GMVs relative to HCs (Created using the BrainNet Viewer Toolbox). (B) The scatter plot shows a correlation between the reduced GMV in the temporal lobe and the off‐ and on‐medication scores for the MDS‐UPDRS III scale. Voxel‐wise significance was set at *p* < 0.001, and cluster‐wise significance was set at *p* < 0.05 (FWE correction). FWE, familywise error; GMV, gray matter volume; HCs, healthy controls; TD, tremor‐dominant Parkinson's disease.

**FIGURE 2:**
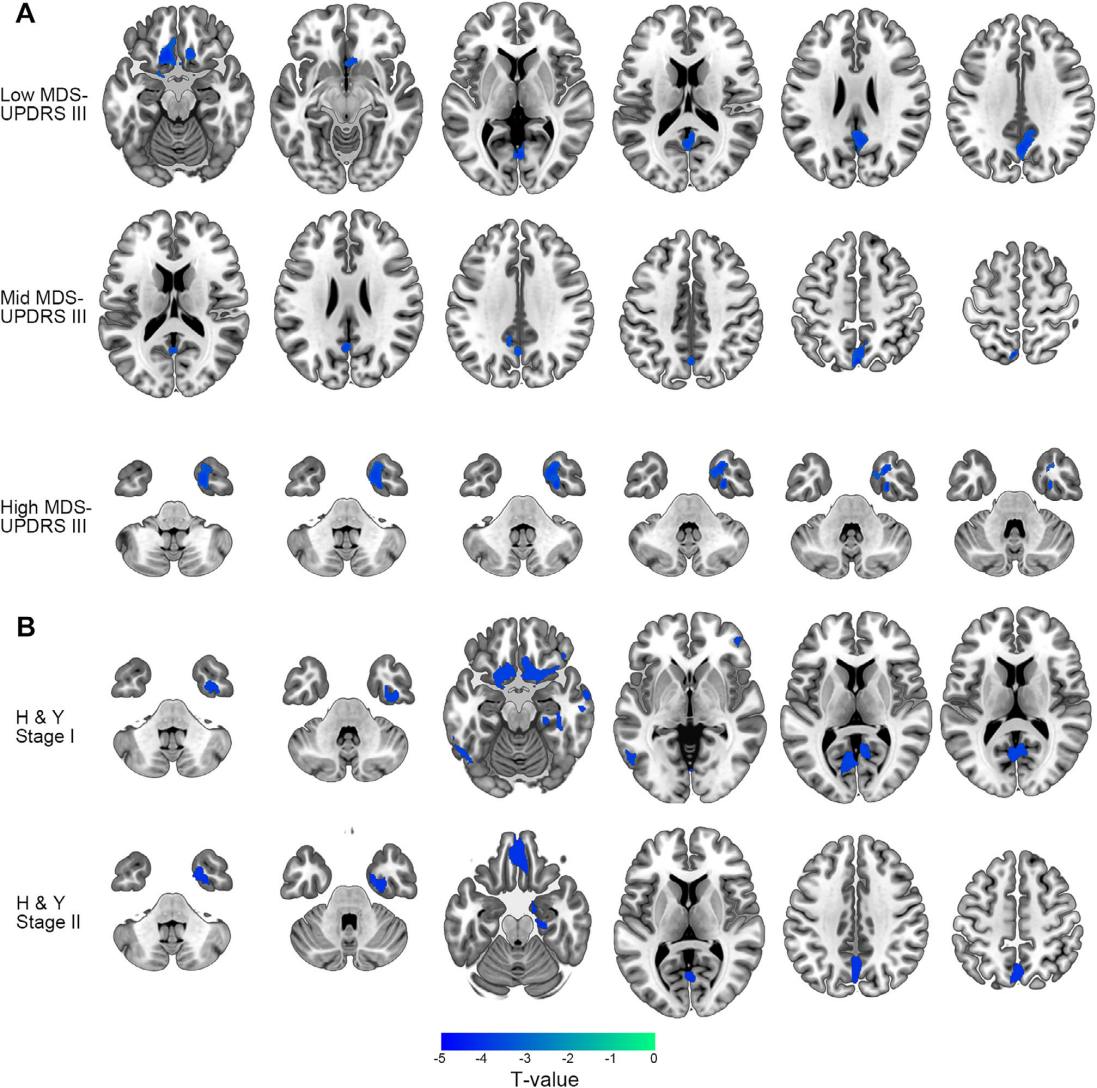
Progressive patterns of decreasing GMV at different stages in patients with TD. All patients were categorized into subgroups by H&Y stages and MDS‐UPDRS III scores. The two grouping methods revealed similar patterns of decreasing GMV, progressively extending from the frontal and parietal lobes to the deep temporal lobe. (A) Brain regions showing significant reductions in GMVs in the low MDS‐UPDRS III score group, mid MDS‐UPDRS III score group, and high MDS‐UPDRS III score group. (B) Brain regions showing significant reductions in GMVs in patients with the H&Y stage I and H&Y stage II. The results were corrected by FWE and are presented with a significance threshold of *p* < 0.05. GMV, gray matter volume; H&Y, Hoehn and Yahr scale; TD, tremor‐dominant Parkinson's disease; UPDRS, Unified Parkinson's Disease Rating Scale.

We performed a voxel‐wise statistical assessment across the entire brain to evaluate GMV alterations between TD patients and HCs. Compared with HCs, TD patients presented significant GMV reductions in the bilateral precuneus (PCUN) of the parietal lobes, left medial orbital frontal gyrus (MOF.L), right fusiform gyrus (FFG.R), and left hippocampus (HIP.L). No significant increases in GMV were observed in TD patients (Figure 1A and Table S1). A subsequent correlation analysis revealed that the FFG.R was related to the on‐medication and off‐medication scores for the third section of the MDS‐UPDRS (MDS‐UPDRS III) (Figure 1B). We then subdivided patients according to their motor symptom severity using MDS‐UPDRS III scores and Hoehn and Yahr (H&Y) stages to characterize the progressive patterns of brain atrophy. Subgroup comparisons based on MDS‐UPDRS III score revealed a distinct pattern of atrophy progression: patients in low MDS‐UPDRS III group primarily exhibited GMV reductions in the bilateral PCUN and left rectus; in mid MDS‐UPDRS III group, atrophy was localized predominantly to the bilateral PCUN; and in high MDS‐UPDRS III group, a further GMV reduction was observed in the FFG.R (Figure 2A). Patients in the H&Y stage I group exhibited early atrophy in the right calcarine (CAL.R), right lingual gyrus (LG.R), right hippocampus (HIP.R), and frontal lobe (right rectus) (Figure 2B). As the disease progressed to H&Y stage II, the GMV decreased in the bilateral PCUN, right parahippocampal region (PHG.R), and frontal lobe (left rectus) (Figure 2B). These results indicated that as the disease progressed, the area of GMV atrophy gradually shifted from the parietal and frontal lobes to the temporal region near the FFG.R. Notably, the bilateral PCUN showed consistent involvement throughout all stages of disease progression, highlighting its potential significance as a central region involved in TD pathology.

### 2.3 Changes in Subcortical Volumes in TD Patients

We subsequently examined changes in the subcortical nuclei among TD patients. Compared with HCs, TD patients presented significant volumetric increases in the bilateral pallidum, white matter (WM) hypointensities, left/right inferior lateral ventricles, and total cerebrospinal fluid (CSF). Additionally, reductions were observed in the bilateral cerebellar cortex, left amygdala, and left accumbens. The details are presented in Table S2.

### 2.4 Structural Covariance Analysis of the PAL.L

**FIGURE 3:**
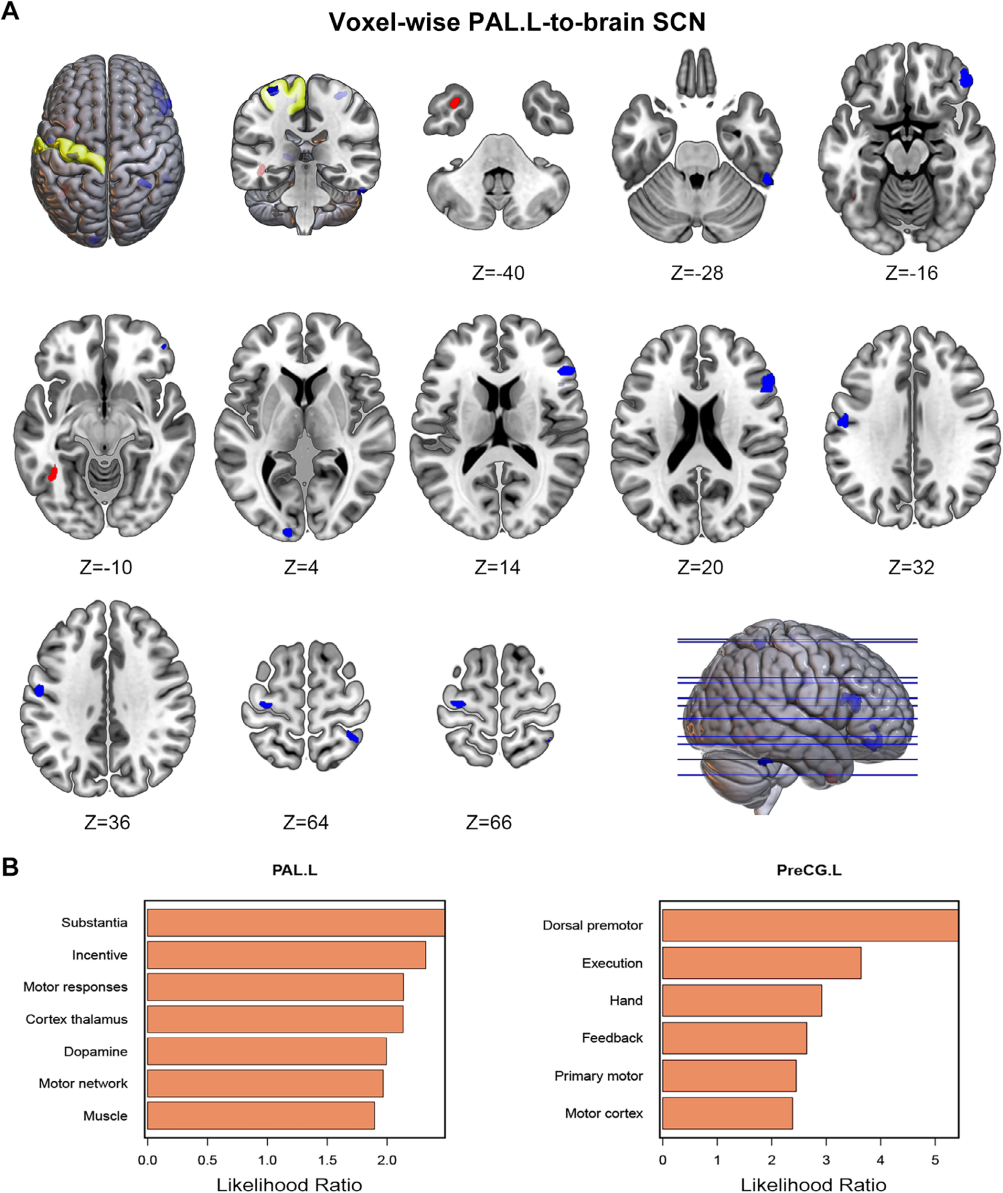
The PAL.L SCN analysis and functional decoding. (A) The SCN was constructed with the left pallidum as the seed region, which showed significant decreases in structural covariance across multiple brain areas, including the anterior cingulate cortex. The blue region indicates decreased GMV covariance, whereas red denotes increased GMV covariance. (B) Functional decoding of the PAL.L and PreCG.L. All reported *p* values are corrected for multiple comparisons. PAL.L, left pallidum; PreCG.L, left precentral gyrus; SCN, structural covariance network.

The left pallidum (PAL.L) was chosen as the regions of interest (ROIs) for the structural covariance analysis because it exhibited the most significant differences. As shown in Figure 3A, the PAL.L‐based structural covariance network (SCN) analysis revealed that TD patients presented a significant increase in GMV covariance in the left fusiform gyrus (FFG.L) and a decrease in GMV covariance across broad cerebral cortices, including the right inferior orbital frontal gyrus (OFG.R), left middle occipital gyrus (MOG.L), right inferior triangular frontal cortex (IFGtriang.R), and left precentral gyrus (PreCG.L). Functional decoding was utilized to investigate the principal functions associated with specific brain regions. The results revealed that the PAL.L was linked primarily to functional keywords such as substantia, incentive, motor responses, cortex thalamus, dopamine, motor network, and muscle. The PreCG.L was mainly related to the keywords dorsal premotor, execution, hand, feedback, primary motor, and motor cortex, which perfectly corresponds to the hand tremor symptoms observed in TD patients (Figure 3B).

### 2.5 Functional Connectivity Analysis and Dynamic Connectivity States

**FIGURE 4:**
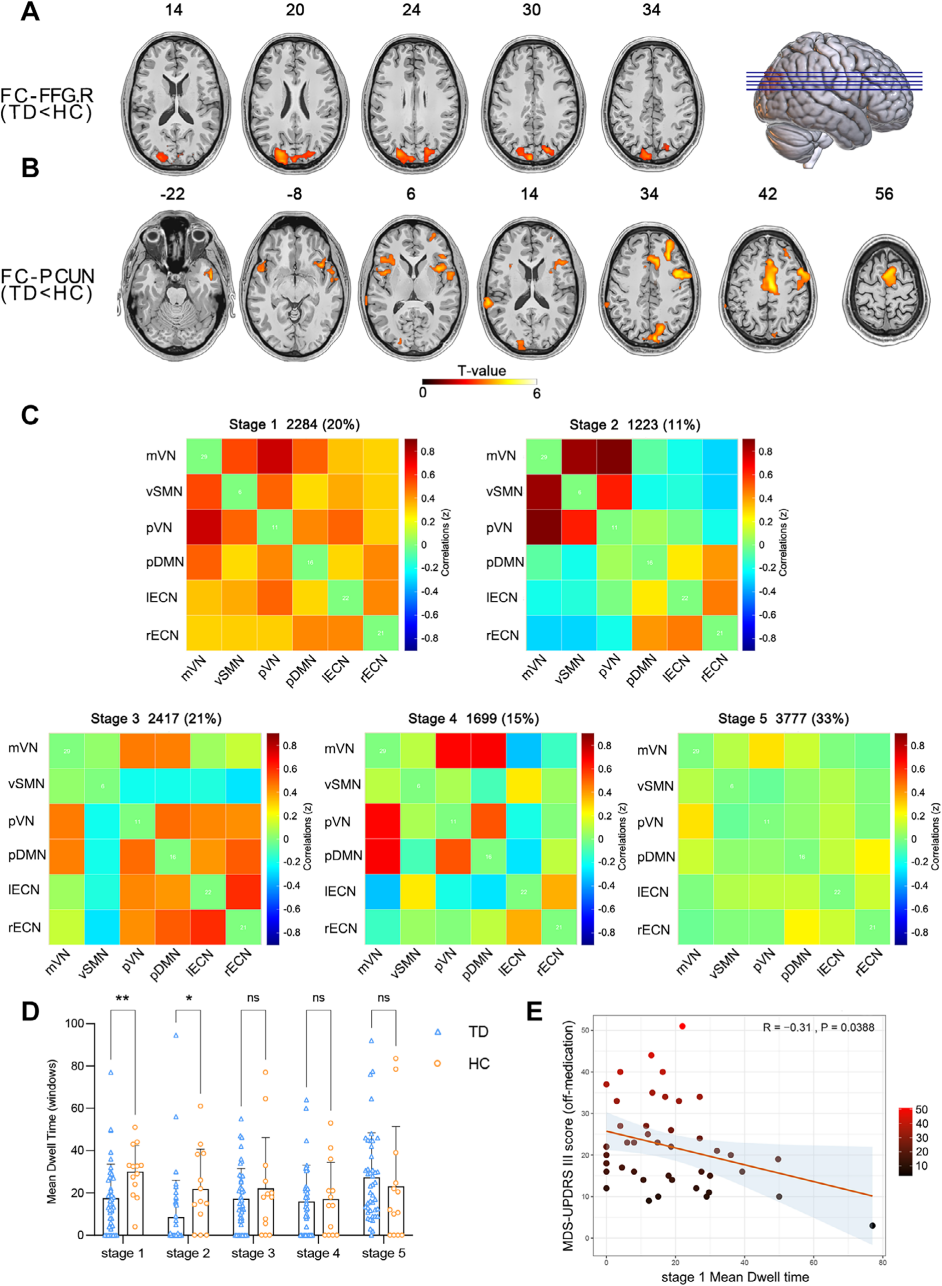
Rs‐fMRI analysis reveals significant differences in FC and dFNC between TD patients and HCs. (A) FC analysis showing that the FC value between the FFG.R and the PCUN was decreased in TD patients compared with HCs. (B) The PCUN exhibited a significant decrease in the FC value across numerous regions of the brain. (C) The dFNC analysis indicated that TD patients could be identified in five different states. (D) The mean dwell times in state 1 and state 2 were significantly shorter in the TD group than in the HCs group (*p < 0.05, **p < 0.01, ns: not significant). (E) The correlation analysis revealed that the mean dwell time in state 1 for the TD group was negatively correlated with the MDS‐UPDRS III score. dFNC, dynamic functional network connectivity; FC, functional connectivity; FFG.R, right fusiform gyrus; HCs, healthy controls; PCUN, precuneus; TD, tremor‐dominant Parkinson's disease.

We performed an ROIs‐wise functional connectivity (FC) analysis using resting‐state fMRI (rs‐fMRI) data from the Parkinson's Progression Markers Initiative (PPMI) database to further explore the functional alterations in the ROIs (FFG.R and PCUN.L) in TD patients. The FC analysis of the FFG.R showed decreased connectivity between the FFG.R and the parieto‐occipital lobe (including the PCUN) in TD patients compared with HCs (Figure 4A). The FC analysis results of the PCUN revealed that the FC of TD patients decreased in a wide range of cortical areas, including the frontal lobe (precentral gyrus, supplementary motor area, middle frontal gyrus, etc.), temporal lobe, and insula (Figure 4B). These findings highlight the central role of the PCUN as a key hub involved in the functional network of TD patients.

Using k‐means clustering, we conducted a dynamic functional network connectivity (dFNC) analysis and identified five characteristic connectivity states in TD patients. Figure 4C presents the connectivity matrices and frequencies of occurrence for each state. Notably, TD patients presented significantly shorter mean dwell times in states 1 and 2 than HCs (Figure 4D). State 1 (strongly connected state), with a frequency of 20%, was characterized by moderate to strong connectivity involving key networks, such as the default mode network (DMN, containing the PCUN), executive control network (ECN), visual network (VN), and sensorimotor network (SMN). State 2, with a frequency of 11%, exhibited strong connectivity predominantly between the SMN and VN, accompanied by generally lower connectivity across other networks. Further analysis revealed that the state 1 mean dwell time in the TD group was negatively correlated with the MDS‐UPDRS III score (*R* = −0.31, *p* = 0.0388) (Figure 4E).

### 2.6 Causal Effects of the GMV Pattern

**FIGURE 5:**
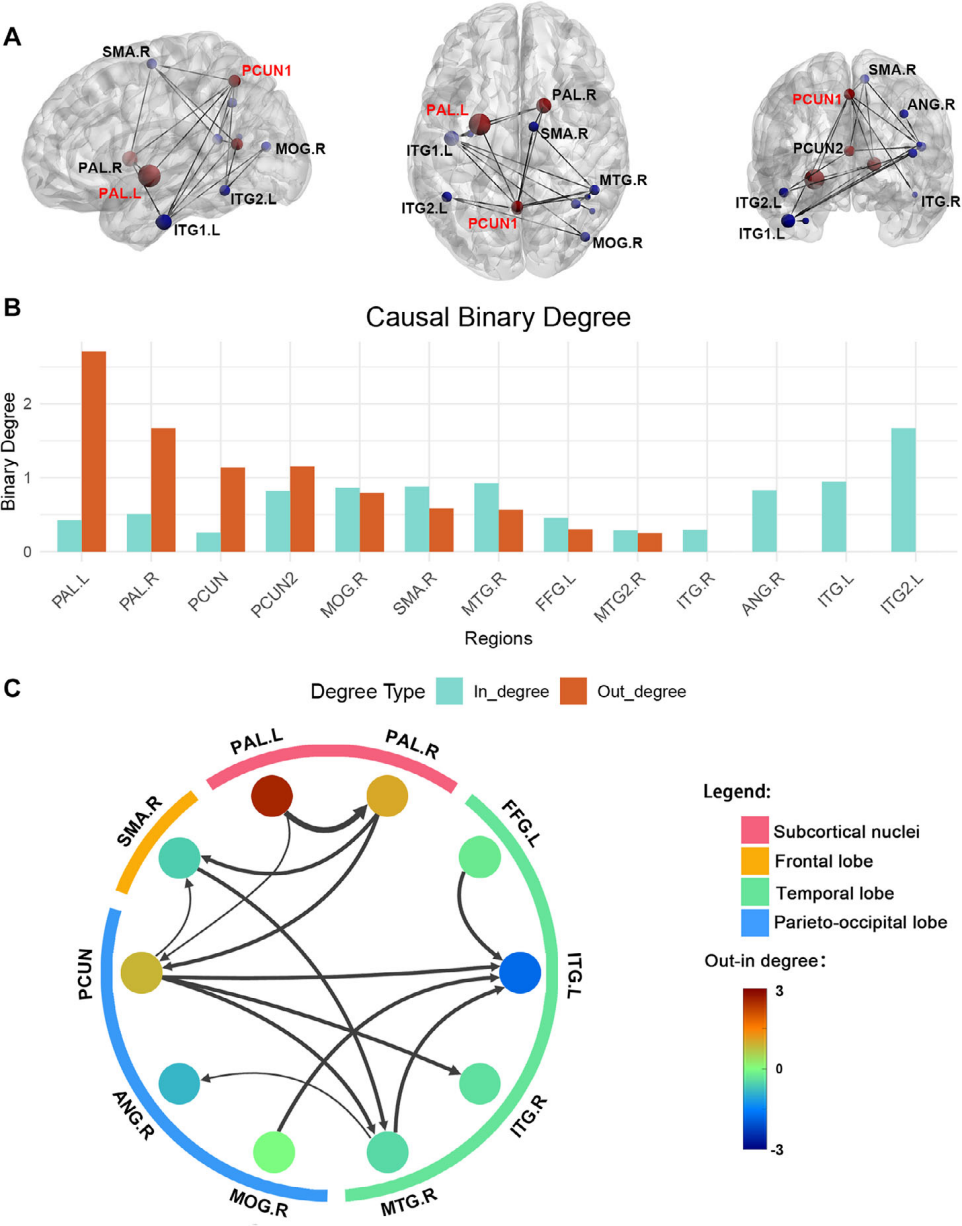
CaSCN showing causal relationships among the brain regions in TD patients. (A) A bivariate signed‐path coefficient Granger causality analysis was performed to construct a regional causal network based on the ROIs (created using the BrainNet Viewer Toolbox). (B) Binary out‐ (orange) and in‐degree (blue) values of each brain region in the CaSCN. (C) The structural covariance causal network of the ROIs was constructed according to the duration of onset. The connections between ROIs represent the direction and magnitude of causal progression and the magnitude of the GC value. CaSCN, causal structural covariance network; GC, Granger causality; ROI, region of interest; TD, tremor‐dominant Parkinson's disease.

We constructed a directed network for the PAL.L and the PCUN by applying GCA to the morphometric data sequenced according to the duration of TD in patients to map the causal effects of the GMV pattern (Figure 5A). The causal binary degree analysis indicated that the PAL.L was the node with the highest out‐degree, directing causal impacts to other regions, while the region in left inferior temporal gyrus had the highest in‐degree, mainly being influenced causally by other regions (Figure 5B). Specifically, the bilateral globus pallidus (PAL.L and PAL.R) predominantly influenced the PCUN regions. The PCUN served as the primary downstream hub regulated by the bilateral globus pallidus and further projected to the bilateral inferior temporal gyrus (ITG.L and ITG.R), right middle temporal gyrus (MTG.R), and right supplementary motor area (SMA.R) (Figure 5C).

### 2.7 Prediction of Surgical Outcomes Based on Brain Morphology

**FIGURE 6:**
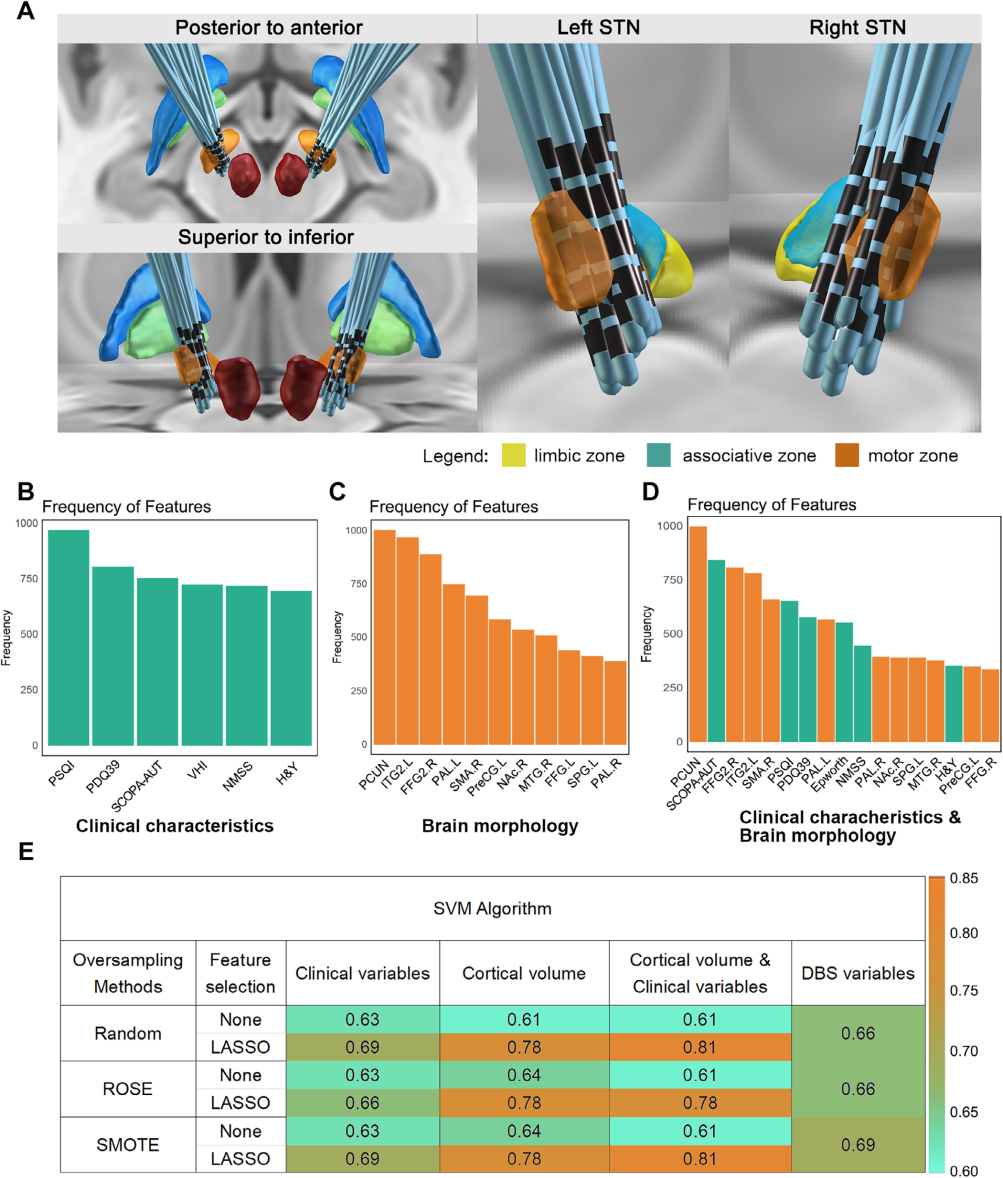
Feature screening and machine learning models based on brain morphology and clinical variables. (A) Schematic illustration of the relationship between the Lead‐DBS group reconstruction results and subregions of the STN. (B) Bar chart showing the importance rankings of clinical features for classification. (C) Bar chart showing the importance rankings of clinical features for brain morphology. (D) Bar chart showing the importance rankings of clinical features for brain morphology and clinical variables. (E) Heatmap of the AUCs of various machine learning models for predicting the tremor type. AUC, area under the curve.

Lead‐DBS software was used to reconstruct electrode positions in patients following DBS treatment (Figure 6A). The reconstruction results showed that the electrodes were inserted into the STN in all patients. The statistical analysis revealed a significant difference in the tremor improvement rate between the STN motor group and the STN associative group (Figure S1A). After the STN was divided into left and right sides, a significant difference in the tremor improvement rate was observed between the right STN (RSTN) motor group and the RSTN associative group (Figure S1B). Additionally, a significant difference in the total levodopa improvement rate was observed between the RSTN motor group and the RSTN associative group (Figure S1C). Moreover, we found distinct correlations between the volume of tissue activated (VTA) within STN subregions and clinical scale scores. Specifically, the VTA volume in the RSTN associative zone was negatively correlated with improvements in the HAM‐A score (*R* = −0.36, *p* = 0.0153) (Figure S1D) and HAM‐D score (*R* = −0.47, *p* = 0.001) (Figure S1E).

We then used linear regression to eliminate the impact of the VTA and classified patients whose postoperative improvement rate was <30% into the poor‐efficacy group. Additionally, feature selection was performed, and the top 30% of features were retained to enhance the model's predictive performance. The feature selection process for each group is shown in Figure 6B–D. In the test cohort, the model that fused brain morphological with clinical variables performed comparably to the models using clinical variables or brain morphology variables alone, considering that the integration model included too many variables. It is worth noting that the prediction performance of the model constructed using VTA alone is also insufficient (Figure 6E). The application of feature filtering markedly enhanced the model's predictive accuracy while reducing the number of variables. With this approach, the integrated model incorporating both brain morphological and clinical features demonstrated superior performance compared to others, achieving an area under the curve (AUC) of 0.78–0.81 (Figure 6E) (Table S3).

## 3 Discussion

In this study, we investigated cortical and subcortical morphological alterations in TD patients through a whole‐brain volumetric analysis. Compared with HCs, TD patients presented significant GMV reductions in the bilateral PCUN, frontal lobe (MOF.L), and bilateral temporal lobe (FFG.R and HIP.L). These results align with earlier research showing cortical atrophy across various brain regions in PD patients, notably involving the superior frontal gyrus, temporal gyri, and PCUN [18]. Other studies also reported significant volumetric reductions in the parietal and temporal lobes in TD patients [19, 20]. The correlation results shown that the FFG.R was negatively correlated with the MDS‐UPDRS III score. Previous research has shown that the GMV in the FFG.R is associated with the latency of visually evoked responses [21]. Recent studies have shown that performing a motor task concurrently with a visual task can reduce the visual processing capacity and visual short‐term memory storage, indicating that motor tasks rely on efficient visual information uptake [22]. These findings may explain why the FFG.R was related to the MDS‐UPDRS III score. We then grouped TD patients based on their H&Y stage and MDS‐UPDRS III scores to further explore the disease progression patterns. As the disease progressed, we observed a marked shift in atrophy patterns from the frontal and parietal lobes to the temporal region. Notably, we observed that the PCUN was involved in all stages of the disease and selected it as an ROI for subsequent analysis.

In addition to cortical GMV changes, we specifically examined the volumes of subcortical nuclei. Our comparative analysis revealed significant alterations in the PAL.L, PAL.R, WM hypointensities, the bilateral inferior lateral ventricles, and total CSF. Among them, the PAL.L showed the most significant difference in terms of the nucleus volume. The pallidum is crucial for voluntary motor regulation and has been linked to key PD symptoms such as tremor and bradykinesia. Using the PAL.L as the seed, the SCN analysis revealed that TD patients presented a significant increase in GMV covariance in the FFG.L region and a decrease in GMV covariance in the PreCG.L and extensive cortical areas. Interestingly, functional decoding revealed that PreCG.L is strongly implicated in hand movement control. Prior research has shown that increased connectivity between the globus pallidus internus and motor cortex contributes to tremor generation in TD patients [23]. Other studies have suggested that functional abnormalities in the basal ganglia may disrupt connectivity within major brain networks, such as DMN, ECN, and SMN [24]. Our findings further confirmed the presence of altered structural connectivity between the pallidum and the motor cortex in TD patients.

We then collected rs‐fMRI data of TD patients from the PPMI database to explore the functional connection patterns of the ROIs. The FC analysis of the FFG.R showed that the FC of the parieto‐occipital lobe (including the PCUN) in TD patients was decreased compared to HCs. The FC analysis of the PCUN showed decreased connectivity with the precentral gyrus, supplementary motor area, and broad frontotemporal regions. These results underscore the pivotal involvement of the PCUN in TD. Atrophy in the PCUN has been noted in patients with different neurodegenerative diseases [25, 26]. An rs‐fMRI study indicated that the PCUN (a component of the DMN) is associated not only with cognitive decline and various nonmotor symptoms but also with motor functions [27]. Another study further reported that a reduced degree centrality in the PCUN was associated with higher UPDRS scores and a longer disease duration in PD patients [28]. The above results suggest that reduced FC between the PCUN and other brain regions may serve as the pathological basis for symptom onset. We conducted a dFNC analysis based on the ROIs identified through the VBM analysis, which were located mainly in the DMN, ECN, and VN, to further investigate the brain network connection pattern. The dFNC analysis clustered the rs‐fMRI data from TD patients into five states, with state 1 being a high‐connectivity state. Compared with that of HCs, the mean dwell time in state 1 was significantly reduced in TD patients, indicating a decreased level of high connectivity between these brain networks. Furthermore, the correlation analysis revealed a negative association between state 1 and MDS‐UPDRS III scores, suggesting that a reduced connectivity strength among these networks is linked to motor symptom severity in TD patients. Mite et al. observed that the directional connectivity patterns among the PCUN, basal ganglia, and cerebellum in PD patients are associated with motor, executive, and memory dysfunction [29]. However, the sequential relationship between the PCUN and the basal ganglia during disease progression remains to be further explored.

A CaSCN analysis can identify core areas of initiation and subsequent susceptible regions, offering a more comprehensive understanding of disease development. In our network, the PCUN functions as the main downstream hub influenced by the bilateral pallidum, subsequently connecting to widespread temporal regions. The PCUN is part of the posterior parietal cortex (PPC), a key area for visual–spatial processing and an associative area that primarily integrates sensory information to guide action [30]. A study of patients with Alzheimer's disease revealed that the PCUN is connected to SMA regions in the paracingulate network via the inferior fronto‐occipital fasciculus, which may help guide motor actions based on the acquisition of new information [31]. Alexandra et al. performed repetitive transcranial magnetic stimulation (rTMS) treatment on the PCUN area in PIGD patients and found that an increase in PPC excitability led to significant reductions in freezing of gait episodes and the duration of freezing [32]. This study further suggested that increased PPC excitability improves visuospatial processing, reduces basal ganglia overload, and helps alleviate symptoms. This evidence suggests that the PCUN may be a potential therapeutic target for noninvasive neuromodulation in PD patients.

For tremor symptoms that are refractory to pharmacological treatment, DBS remains the most commonly used and effective invasive therapy for controlling motor symptoms in PD patients [33]. However, individual sensitivity to DBS varies, and predicting this difference remains clinically challenging. Our goal was to develop a machine learning model that could effectively discriminate patients’ sensitivity to DBS utilizing brain morphology data. A statistical analysis of electrode reconstruction in TD patients revealed that electrodes in the associative zone resulted in a better tremor improvement rate than those in the motor zone. Additionally, we found that the VTA in the associative zone of the STN was negatively correlated with clinical improvements measured by both the HAM‐A and the HAM‐D. These findings are consistent with those of previous studies [34, 35]. We subsequently employed linear regression to eliminate the confounding effects of VTA on improvement rates. We further utilized the support vector machine (SVM) model based on morphology imaging to predict patients’ postoperative improvement rate. In this model, the VTA alone was not a good predictor of DBS sensitivity. Applying feature filtering further increased predictive accuracy while reducing the number of variables. Integrating brain morphology with clinical variables enhanced model performance compared with either data type alone. Our results suggested that combining imaging and clinical data can effectively predict DBS responsiveness and serve as a practical tool for precision medicine.

Notwithstanding the significance of these findings, certain limitations of this study must be recognized. First, the CaSCN analysis was based on a pseudotime series derived from patient onset times, which can suggest only the extent of causal influence but does not directly reflect the actual temporal progression of TD. Second, although rs‐fMRI data were obtained from the publicly available PPMI database to increase data robustness, the lack of matching between structural MRI and functional MRI datasets may introduce variability. Finally, the sample size in this study remains relatively modest. Larger‐scale, multicenter cohorts are needed to confirm the robustness and broaden the generalizability of our findings.

## 4 Conclusions

This study comprehensively investigated the structural changes in the cerebral cortex and subcortical nuclei in TD patients and identified the pallidum and PCUN as critical areas associated with early cortical damage. As the disease duration in TD patients increases, GMV changes extend from the PAL.L to the PCUN and then to the whole brain, indicating that the disease duration is related to structural brain changes during TD progression.

## 5 Methods and Materials

### 5.1 Patients

The diagnosis of PD was made by physicians according to the International Parkinson and Movement Disorder Society (MDS) Clinical Diagnostic Criteria for Parkinson's Disease [36]. According to the clinical phenotypes, the prototypic PD patients were classified into the TD subtype and the PIGD subtype. In the MDS‐UPDRS calculations (first column), the ratio of the mean tremor score to the mean PIGD score is used to classify clinical phenotypes into TD and PIGD [37]. Patients with a ratio ≥ 1.15 were classified as TD, whereas patients with a ratio <0.9 were classified as PIGD [38]. Sixty‐one patients with TD were recruited from January 2018 to May 2023. Sixty‐one HCs matched by age, sex, and educational background were selected from the Physical Examination Center at Beijing Tiantan Hospital. The inclusion criteria for PD patients were as follows: (1) met the MDS clinical diagnostic criteria with an MDS‐UPDRS TD/PIGD ratio ≥ 1.15; (2) had been treated with levodopa and/or dopamine agonists for at least 6 months; (3) had MRI results that met the required standards; (4) had a Mini‐Mental State Examination (MMSE) score ≥ 24; and (5) had not undergone any surgical treatment before admission. Patients whose conditions resulted from stroke, pharmacological factors, encephalitis, toxic insults, traumatic injury, or other degenerative neurological diseases, as well as those with a history of dementia or epilepsy, were excluded. The control participants were examined by two neurosurgeons who were blinded to the MRI results to exclude any neurological disease, such as stroke, tumor, or severe multifocal ischemic leukoencephalopathy.

### 5.2 Clinical Assessment

Prior to MRI scans, baseline demographic information (age, sex, education level, and disease duration) and clinical measures such as H&Y stage [39], VHI score [40], MDS‐UPDRS score, PDQ39, PSQI, the Scales for Outcomes in Parkinson's disease‐Autonomic (SCOPA‐AUT), Non‐Motor Symptoms Scale (NMSS), and total levodopa equivalent dose (LED) were collected. The cognitive status of TD patients was assessed using the MMSE [41] and the Montreal Cognitive Assessment (MoCA) [42]. Affective disorder states were evaluated by HAM‐A [43] and HAM‐D [44]. Motor symptoms, including tremor, were evaluated in TD patients using part III of the MDS‐UPDRS scale. Before testing, patients were confirmed to have not taken levodopa or other anti‐Parkinsonism medications for at least 12 hours, after which their off‐medication scores were recorded. The on‐medication assessment was strictly conducted according to the standardized levodopa challenge test [45]. Briefly, the on‐medication score was recorded at the peak of therapeutic efficacy after the patient received 1.5 times their usual morning dose of levodopa. Postoperative assessments were conducted at 12 months following surgery. All PD patients were categorized into subgroups based on disease severity to evaluate the progressive pattern of GMV atrophy. Patients were divided into two subgroups according to the H&Y scale score: H&Y stage I (scores ranging from 1 to 2.5) and H&Y stage II (scores ranging from 2.5 to 4), with no patients classified as H&Y stage 5 in the TD group. Additionally, patients were divided into three subgroups according to tertiles of their MDS‐UPDRS III scores: the first tertile included scores ≤ 38, the second tertile included scores between 40 and 51, and the third tertile included scores > 51.

### 5.3 Image Acquisition

All TD patients and HCs underwent a 3D T1‐weighted MRI scan at Beijing Tiantan Hospital. The computed tomography (CT) imaging data of TD patients were collected on the day after surgery to aid in the reconstruction of electrodes and the VTA. The detailed parameters were as follows: 3D T1‐weighted MRI using a Siemens Prisma 3.0 T scanner; repetition time (TR) = 1560 ms, echo time (TE) = 1.69 ms, matrix size = 256 × 256 mm^2^, flip angle (FA) = 8, field of view (FOV) = 240 mm, scanning time = 5 min 53 s, 192 slices, and voxel size = 1.0 × 1.0 × 1.0 mm^3^. Standard CT used a Philips CT scanner with 304 slices and a voxel size = 0.4833 × 0.4833 × 0.625 mm^3^.

To further explore the FC between the ROIs, rs‐fMRI data were collected from the PPMI database (http://www.ppmi‐info.org). All the rs‐fMRI data were collected using the same machine model and scanning parameters to ensure the reliability of the results. A total of 44 TD patients (31 males and 13 females) who met the diagnostic criteria were included, along with 13 matched HCs (nine males and four females). The mean age of TD patients was 64.4 ± 6.27 years, while that of the HCs was 63.5 ± 6.44 years. A 3.0 T Siemens scanner was used for image acquisition. The rs‐fMRI data parameters were as follows: FA = 80, matrix X = 448 pixels, matrix Y = 448 pixels, pixel spacing X = 3.5 mm, pixel spacing Y = 3.5 mm, TR = 2500 ms, TE = 30 ms, and slice thickness = 3.5 mm. The 3D T1‐weighted images matched to the rs‐fMRI scans were acquired with the following parameters: FA = 90, matrix X = 256 pixels, matrix Y = 256 pixels, matrix Z = 192, pixel spacing X = 1 mm, pixel spacing Y = 1 mm, slice thickness = 1.0 mm, TE = 3.0 ms, and TR = 2300 ms.

### 5.4 VBM Analysis

Preprocessing of the initial 3D T1‐weighted images was performed by MRIcroGL, and then the images were resized to an appropriate size. In accordance with the standard VBM computational workflow used in previous studies [46], the images were subsequently processed with the CAT12 toolbox within the Statistical Parametric Mapping (SPM12, https://www.fil.ion.ucl.ac.uk/spm) tool in MATLAB software. All the filtered GM images were smoothed using an 8 × 8 × 8 mm^3^ full‐width‐at‐half‐maximum (FWHM) Gaussian kernel. A general linear model was developed that incorporated sex, age, and total intracranial volume (TIV) as covariates to compare differences in GMVs between the TD and HCs groups. SCN analysis is a practical tool for assessing morphological synchronization between different areas of the brain and can reveal brain connectivity and structural networks affected by various diseases [47]. The data were corrected for multiple comparisons using family‐wise error (FWE), and the threshold was set at *p* < 0.05.

### 5.5 Analysis of Subcortical Structures

The resized 3D T1‐weighted images obtained in the previous step were processed using the stable version of FreeSurfer (version 7.1.1, http://surfer.nmr.mgh.harvard.edu). As the details of the FreeSurfer analysis process have been extensively documented in prior publications, they are not reiterated here [48, 49]. These volumes were compared between TD patients and HCs using *t* tests, with sex, age, and TIV included as covariates. Multiple comparisons were adjusted using the false discovery rate (FDR), and significance was defined as *p* < 0.05.

### 5.6 Rs‐fMRI Preprocessing

The rs‐fMRI datasets were processed with the RESTplus toolbox (version 1.27, https://www.restfmri.net/). Preprocessing of rs‐fMRI data was carried out according to standard procedures detailed in previous literature [50]. The FC results were adjusted using FDR correction with thresholds of *p* < 0.005 (voxel level) and *p* < 0.05 (cluster level).

### 5.7 The dFNC Analysis

The dFNC analysis was estimated using the sliding window method of the GIFT toolbox, as described in previous publications [51]. After data preprocessing, the independent component analysis (ICA) method was implemented using GIFT software to analyze the data from all the subjects [52]. Among the 30 components generated by ICA, we combined previous brain morphology studies and selected six ICA components that summarized the four functional networks most relevant to PD, the SMN, VN, DMN, and ECN, as the focus of subsequent analysis (Figure S2). We used a sliding window approach to calculate the dFNC for the ICA procedure [53]. We examined the differences in dFNC states between groups by evaluating the mean dwell time, which measures the average continuity within the same state.

### 5.8 CaSCN Analysis

GCA is a statistical method that assesses whether one time series can predict the future values of another time series [54]. The CaSCN analysis integrates the SCN analysis with GCA to investigate the temporal changes in structural relationships between different brain regions and to deduce potential causal links. Consistent with other VBM‐based CaSCN studies, we developed a seed‐based CaSCN in which seed regions were identified based on previous VBM and SCN results [15]. Specifically, all GMV data from TD patients were ordered by the duration of the disease, which were utilized as “pseudotime series” data to depict the progression of TD. Sex, age, and TIV were considered covariates in the analysis. Only positive GCA values were retained to forecast structural changes in causality between ROIs. All positive “seed‐to‐map” values were transformed into z scores, with thresholds meeting the FDR criteria (*p* < 0.05). GCA among the ROIs was also performed to explore their causal interactions using the same GC value thresholds. The causal network created using BrainNet Viewer Toolbox (https://www.nitrc.org/projects/bnv/). Finally, we calculated “out‐degree” and “in‐degree” values for every ROI to examine the sequential patterns of causal changes across the regions.

### 5.9 Functional Decoding

We conducted a functional characterization analysis using reverse inference methods based on the BrainMap database to objectively characterize the functions of the identified ROIs [14]. This approach was employed to determine the behavioral domains associated with the selected ROIs. The significance threshold was set at *p* < 0.05, and the results were corrected using the FDR method.

### 5.10 Lead‐DBS Electrode Reconstruction

The surgical procedure was as follows: One day before surgery, patients underwent a high‐resolution MRI scan, and on the following morning, a stereotactic head CT scan was obtained with the Leksell stereotactic frame (Elekta Inc., Sweden). The electrodes were implanted after combining the CT and MRI scans for target localization using the Leksell‐SurgiPlan (version 10.1) surgical system [55]. The target coordinates for the STN were set as follows: *X* = 11.0–13.0 mm (lateral to the midline), *Y* = −1.0 to −3.0 mm (posterior to the midpoint of the anterior commissure–posterior commissure [AC‐PC] line), and *Z* = −4.0 to −6.0 mm (below the AC‐PC plane). In this study, imaging data from TD patients who underwent bilateral STN‐DBS surgery were collected and processed with Lead‐DBS (version 2.6, http://www.lead‐dbs.org), following a methodology similar to that used in previous research [35]. This analysis of the spatial distribution of the VTA allowed us to establish the relationship between the placement of DBS electrodes and STN subregions and to categorize TD patients into subgroups based on the extent of VTA coverage within these subregions. Initially, patients were categorized into two groups: the sensorimotor zone group (*n* = 31) and the associative zone group (*n* = 20). We further elucidated the impact of electrode placement in different cerebral hemispheres and their relationships with STN subregions with symptom improvement by dividing each group into two subgroups according to hemisphere: the left sensorimotor zone group, left associative zone group, right sensorimotor zone group, and right associative zone group. Finally, we used the following formula to calculate the improvement rate of scores on clinical scales such as the HAM‐A and HAM‐D after surgery: improvement rate = [(baseline score − postsurgery score)/baseline score] × 100%.

### 5.11 Feature Selection and Multimachine Learning

Fifty‐one TD patients treated with bilateral STN‐DBS were included, and the dataset was randomly partitioned into training and test sets at a 3:1 ratio. Least absolute shrinkage and selection operator (LASSO) regression was applied for feature selection with the “glmnet” package in R, and repeated 1000 times on the training set. An SVM‐based machine learning approach was employed to estimate patient outcomes. For each of 1000 iterations, predicted probabilities were obtained for individuals in the test cohort and used to derive corresponding AUC metrics. We computed 1000 AUC values as performance metrics for each model, with the lower and upper 2.5 percentile values serving as benchmarks. By considering 1000 iterations of the model, we determined the overall performance based on the median AUC.

### 5.12 Statistical Analysis

Statistical analyses were performed using GraphPad Prism 10 (San Diego, CA, USA) and R software (version 4.4.2). Pearson correlation analysis was used to assess the relationships between cortical volume and clinical scores, with age and sex included as covariates. Statistical significance was defined as a *p* value < 0.05. For each of the connectivity states, we compared mean dwell times using Mann–Whitney *U* tests. Results are presented as mean ± standard deviation (SD), and an FDR‐corrected *p* < 0.05 was considered statistically significant.

## Author Contributions

M.X.Z., S.Y.Z., and P.D.Y. conceived the study, completed the data analysis, wrote the manuscript, and are the guarantors of the work. H.Z.W. contributed to the collection of experimental data and played a significant role in the revision of the manuscript. J.L.D., X.B.W., and X.Z.C. supported the acquisition of imaging data and provided insightful advice regarding data analysis. C.N.Z., A.N.W., and Y.G. obtained funding. Q.L., Y.C.J., Y.J., L.S., and C.L.H. provided advice on the statistical analyses. Z.Y., T.F., J.G.Z., and F.G.M. contributed to the methodology and provided guidance on writing the manuscript. All the authors contributed to reviewing and approving the final manuscript.

## Ethics Statement

This study was approved by the Ethics Committee of Beijing Tiantan Hospital (No. KYSQ 2022‐389‐01‐01). The research complied with the Declaration of Helsinki, and all participants provided written informed consent.

## Conflicts of Interest

The authors declare no conflicts of interest.

## Supporting information




**Figure S1**: Results of the Lead‐DBS group analysis compared with the clinical scale scores. All patients with TD were categorized into sensorimotor or associative groups based on the volume of the STN subregions affected by the VTA. (A‐C) Significant changes in symptom improvement rates across different subgroups (*p < 0.05). (D‐E) A significant correlation was observed between VTA and the clinical rating scales.
**Figure S2**: Spatial maps of independent components were selected as our networks of interest.
**Table S1**: Significant cortical volume differences in TD compared to HCs from voxel‐wise analysis.
**Table S2**: Cerebral nuclei volumes of each group and *p* value of group comparisons between TD and HCs.
**Table S3**: Performances of the SVM models with feature selection in the test set.

## Data Availability

Beijing Tiantan Hospital has an institutional commitment to data sharing. These data are available from the corresponding author upon reasonable request.
