# Supplementary material for: The Precuneus Region Drives Brain Network Changes in Tremor‐Dominant Parkinson's Disease: Insights from a Morphological Causal Analysis
Source: MedComm (2020). 2025 Oct 26;6(11):e70441. doi: 10.1002/mco2.70441 (PMC12554785; doi:10.1002/mco2.70441)
Supplement: Supplementary file 1 — Figure S1: Results of the Lead‐DBS group analysis compared with the clinical scale scores. All patients with TD were categorized into sensorimotor or associative groups based on the volume of the STN subregions affected by the VTA. (A‐C) Significant changes in symptom improvement rates across different subgroups (*p < 0.05). (D‐E) A significant correlation was observed between VTA and the clinical rating scales. Figure S2: Spatial maps of independent components were selected as our networks of interest. Table S1: Significant cortical volume differences in TD compared to HCs from voxel‐wise analysis. Table S2: Cerebral nuclei volumes of each group and p value of group comparisons between TD and HCs. Table S3: Performances of the SVM models with feature selection in the test set. [file MCO2-6-e70441-s001.pdf]

Supplementary material

The precuneus region drives brain network changes in tremor-dominant Parkinson's disease: insights from a morphological causal analysis

Moxuan Zhang[1,2,#], Siyu Zhou[1,3,#], Pengda Yang[1,3,#], Huizhi Wang[1,3,#], Jinli Ding[4], Xiaobo Wang[4], Xuzhu Chen[4], Chaonan Zhang[1,3], Anni Wang[1,3], Yuan Gao[1,3], Qiang Liu[1,3], Yuchen Ji[5],Yin Jiang[1], Lin Shi[3], Chunlei Han[3], Zhong Yang[6*], Tao Feng[7*], Jianguo Zhang[3*], Fangang Meng[1,3,5*]

[1]Beijing Neurosurgical Institute, Capital Medical University, Beijing, China
[2]Department of Neurosurgery, Shandong Cancer Hospital and Institute, Shandong First Medical University and Shandong Academy of Medical Sciences, Jinan, China
[3]Department of Neurosurgery, Beijing Tiantan Hospital, Capital Medical University, Beijing, China
[4]Department of Radiology, Beijing Tiantan Hospital, Capital Medical University, Beijing, China
[5]Department of Neurosurgery, The First Affiliated Hospital of Zhengzhou University, Zhengzhou, Henan, China
[6]Department of Humanities and Social Sciences, Binzhou Medical University, Shandong, China
[7]Department of Neurology, Beijing Tiantan Hospital, Capital Medical University, Beijing, China

***Correspondence**

Zhong Yang, Department of Humanities and Social Sciences, Binzhou Medical University, Shandong 264003, China.
E-mail: yangzhong@bzmu.edu.cn
Tao Feng, Department of Neurology, Beijing Tiantan Hospital, Capital Medical University, Beijing 100070, China.
E-mail: bxbkyjs@sina.com
Jianguo Zhang, Department of Neurosurgery, Beijing Tiantan Hospital, Capital Medical University, Beijing 100070, China.
E-mail: jianguozhang@ccmu.edu.cn
Fangang Meng, Beijing Neurosurgical Institute, Capital Medical University, Beijing 100070, China.
E-mail: fgmeng@ccmu.edu.cn

# Moxuan Zhang, Siyu Zhou, Pengda Yang and Huizhi Wang contributed equally to this work and shared first authorship.

FIGURE S1 Results of the Lead-DBS group analysis compared with the clinical scale scores. All patients with TD were categorized into sensorimotor or associative groups based on the volume of the STN subregions affected by the VTA. (A-C) Significant changes in symptom improvement rates across different subgroups (**p* < 0.05). (D-E) A significant correlation was observed between VTA and the clinical rating scales.

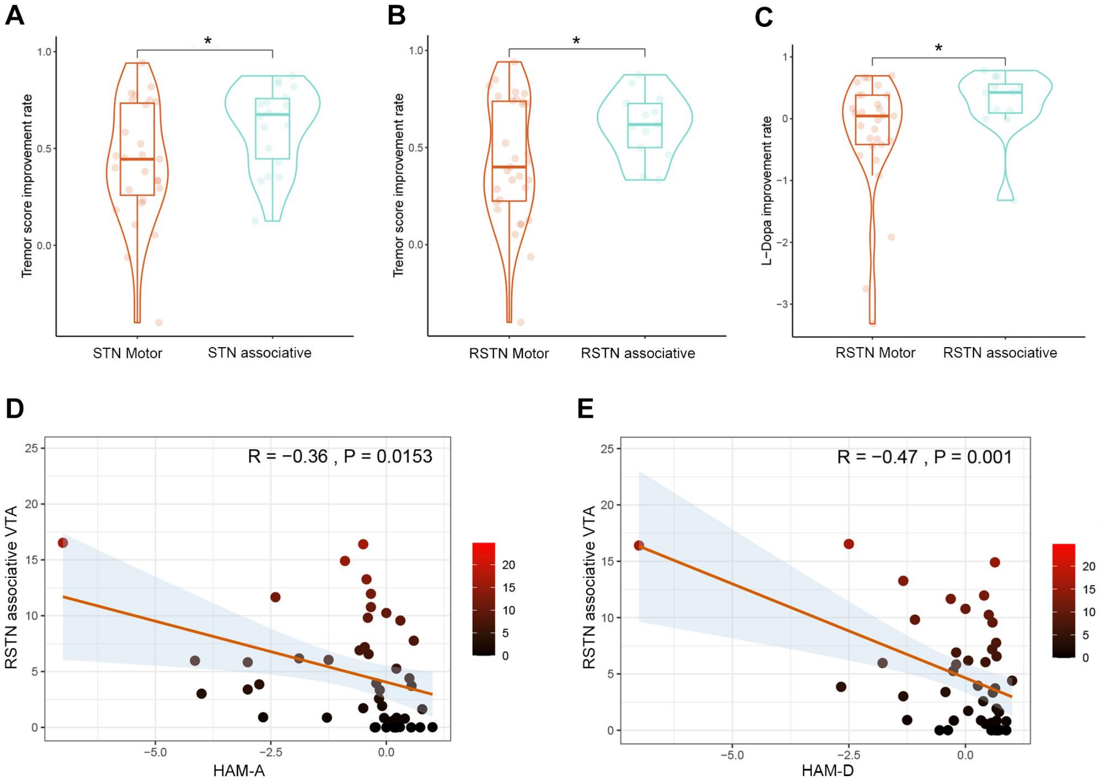

DBS, deep brain stimulation; STN, subthalamic nucleus; VTA, volume of tissue activated.

FIGURE S2 Spatial maps of independent components were selected as our networks of interest.

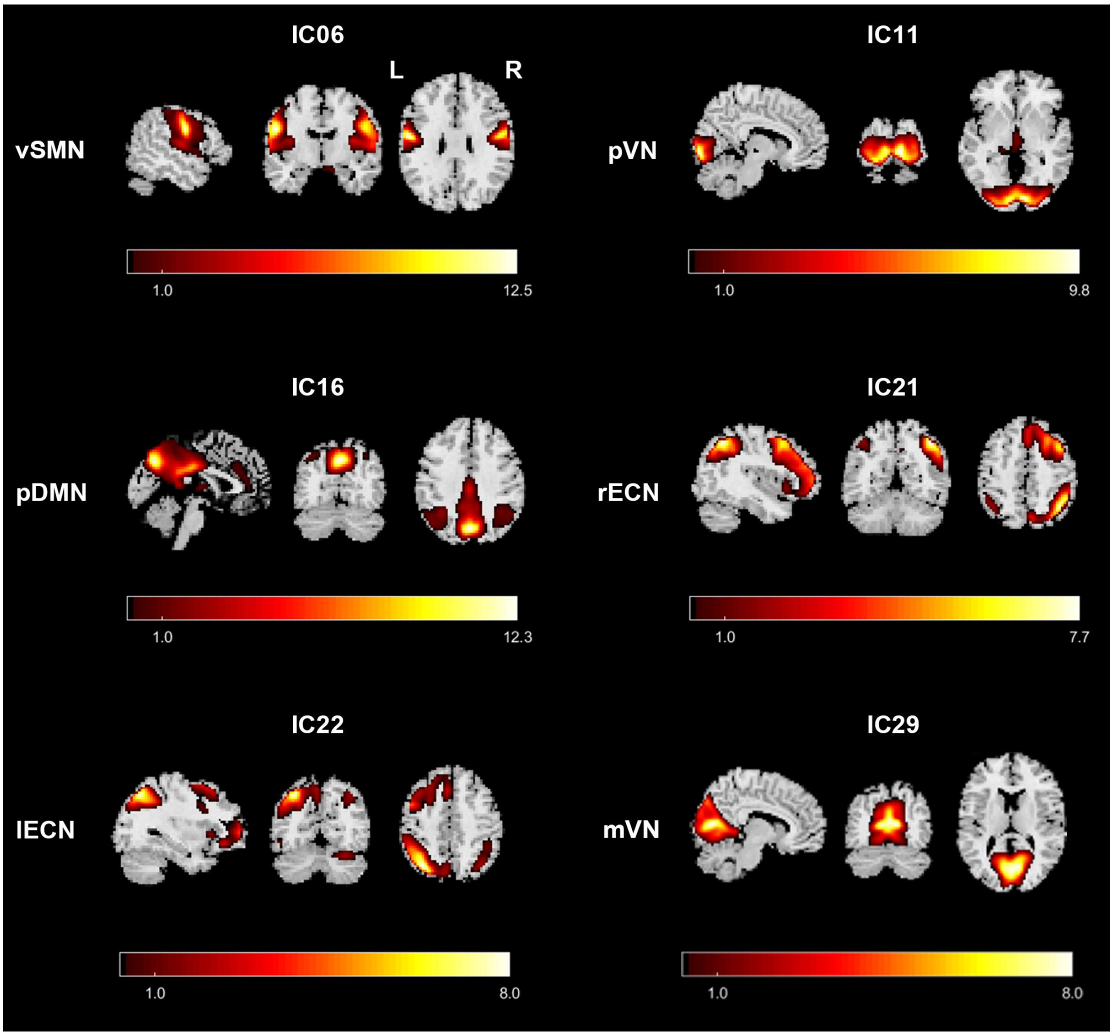

L, left; R, right; vSMN, ventral sensorimotor network; pVN, posterior visual network; pDMN, posterior default mode network; rECN, right executive control network; lECN, left executive control network; mVN, medial visual network.

TABLE S1 Significant cortical volume differences in TD compared to HCs from voxel-wise analysis.

Regions	Abbreviation	MNI coordinates			T value	Cluster Size	*p* value
		X	Y	Z			
Precuneus	PCUN	-1.5	-75	52.5	5.40	3531	<0.001
Left medial orbital gyrus	MOF.L	-1.5	63	-13.5	4.96	2735	<0.001
Right fusiform	FFG.R	33	-4.5	-39	5.31	2120	0.001
Left hippocampus	HIP.L	-25.5	-12	-12	4.95	2084	0.001

voxel $p<0.001$, cluster $p<0.05$, FWE corrected.

TABLE S2 Cerebral nuclei volumes of each group and P value of group comparisons between TD and HCs.

Nuclei	HCs		TD		FDR
	Mean	SD	Mean	SD	*p* value
Left Pallidum	1864.62	220.06	2057.27	285.77	0.003
Right Cerebellum Cortex	53175.77	4572.36	51091.75	5615.51	0.004
Left Cerebellum Cortex	51497.61	4613.09	49672.06	5020.38	0.013
Left Amygdala	1663.02	230.02	1571.06	238.57	0.013
WM Hypointensities	1179.45	754.71	2519.93	2525.86	0.016
Left Inf Lat Vent	428.97	224.38	653.98	410.41	0.019
Right Pallidum	1837.41	231.19	1988.67	261.38	0.019
Right Lateral Ventricle	9659.92	4088.65	13321.73	6479.35	0.025
Right Inf Lat Vent	430.62	220.14	617.16	353.41	0.026
CSF	1101.80	252.18	1259.76	288.47	0.032
Left Accumbens	453.77	97.11	418.03	88.35	0.033
Left Lateral Ventricle	11088.83	5025.78	15514.02	8354.79	0.040

TABLE S3 Performances of the SVM models with feature selection in the test set.

SVM Algorithm					
Oversampling Methods	Feature selection	Clinical variables Median AUC (95% CI)	Cortical volume Median AUC (95% CI)	Cortical volume & Clinical variables Median AUC (95% CI)	DBS variables Median AUC (95% CI)
Random	None	0.63 (0.44, 0.91)	0.61 (0.42, 0.92)	0.61 (0.42, 0.89)	0.66 (0.44, 0.94)
	LASSO	0.69 (0.44, 0.97)	0.78 (0.50, 1.0)	0.81 (0.53, 1.0)	
ROSE	None	0.63 (0.44, 0.91)	0.64 (0.44, 0.92)	0.61 (0.45, 0.89)	0.66 (0.44, 0.91)
	LASSO	0.66 (0.44, 0.94)	0.78 (0.47, 1.0)	0.78 (0.50, 1.0)	
SMOTE	None	0.63 (0.44, 0.88)	0.64 (0.42, 0.89)	0.61 (0.42, 0.89)	0.69 (0.44, 0.94)
	LASSO	0.69 (0.47, 0.97)	0.78 (0.53, 1.0)	0.81 (0.53, 1.0)	
